# Identification of SARS-CoV-2-against aptamer with high neutralization activity by blocking the RBD domain of spike protein 1

**DOI:** 10.1038/s41392-021-00649-6

**Published:** 2021-06-10

**Authors:** Ge Yang, Ziyue Li, Irfan Mohammed, Liping Zhao, Wei Wei, Haihua Xiao, Weisheng Guo, Yongxiang Zhao, Feng Qu, Yuanyu Huang

**Affiliations:** 1 School of Life Science; Advanced Research Institute of Multidisciplinary Science; Institute of Engineering Medicine; Key Laboratory of Molecular Medicine and Biotherapy, Beijing Institute of Technology, Beijing, China; 2 State Key Laboratory of Biochemical Engineering, Institute of Process Engineering, Chinese Academy of Sciences, Beijing, China; 3 Beijing National Laboratory for Molecular Sciences, State Key Laboratory of Polymer Physics and Chemistry, Institute of Chemistry, Chinese Academy of Sciences, Beijing, China; 4 Translational Medicine Center, Key Laboratory of Molecular Target & Clinical Pharmacology and the State Key Laboratory of Respiratory Disease, School of Pharmaceutical Sciences & the Second Affiliated Hospital, Guangzhou Medical University, Guangzhou, China; 5 National Center for International Research of Biological Targeting Diagnosis and Therapy, Guangxi Key Laboratory of Biological Targeting Diagnosis and Therapy Research, Guangxi Medical University, Nanning, Guangxi China

**Keywords:** Nucleic-acid therapeutics, Drug screening, Molecular medicine, Therapeutics, Infectious diseases

**Dear Editor,**

The ongoing outbreak of coronavirus disease 2019 (COVID-19) caused by severe acute respiratory syndrome coronavirus-2 (SARS-CoV-2) poses a great threat to the public health of people and the normal economic and social development around the world. As of January 8, 2021, more than 88 million people were infected with SARS-CoV-2, resulting in more than 1.9 million death. Early detection and treatment of SARS-CoV-2 are of prime significance for effective control of COVID-19. Currently, nucleic acid amplification (e.g., RT-PCR) and detection of IgG, IgM, or viral antigen are employed in clinical diagnosis^1^, and there is only one drug, remdesivir, was approved for COVID-19 treatment. Although various virus-based and host-based therapeutics, such as remdesivir, lopinavir/ritonavir, umifenovir, ribavirin, chloroquine, hydroxychloroquine, interferon, Tocilizumab, convalescent plasma, neutralizing antibody, and traditional Chinese medicine, have been tested to treat COVID-19^2^, developing novel therapeutic are urgently required. Nucleic acid modalities including aptamer oligonucleotide constitute a promising next-generation solution for disease diagnosis and treatment^3–5^. Developing an aptamer-based theranostic regimen represents a potentially effective way to control COVID-19.

We herein report that six novel DNA aptamers, screened via capillary electrophoresis (CE)-based systematic evolution of ligands by exponential enrichment (SELEX) method, can specifically recognize SARS-CoV-2 in human serum, potently inhibit SARS-CoV-2 infection by blocking the receptor-binding domain (RBD) domain of spike (S) protein subunit 1. The spike protein composed of S1 and S2 subunits, mediating the recognition and infection of SARS-CoV-2. Particularly, the S1 subunit-containing RBD is an important site for host neutralizing antibodies and a promising target for vaccine design. It can interact with the angiotensin-converting enzyme (ACE2) to infect human respiratory epithelial cells. Hence, we screened S1 protein against DNA aptamers, aiming to achieve specific detection and/or effective inhibition of SARS-CoV-2 infection.

SELEX of oligonucleotide aptamers of active virulent viruses has extremely high safety requirements for separation equipment and environment. Therefore, in order to increase the feasibility of screening strategies, recombinant S1 protein was thrown to capture the entire SARS-CoV-2. In order to ensure the evolution efficiency of the affinity and specificity of aptamer candidates, we adopted a combined SELEX strategy of the positive, negative, and complex background screening process, and continuously reduced the concentration of S1 to increase the screening pressure to obtain high-performance aptamers (Supplementary Fig. S1). In the final round of screening, the evolved sub-library bound to S1 protein to form a stable complex in human serum. After high-throughput sequencing, the six most enriched aptamer candidates (nCoV-S1-Apts) were selected for performance verification (Supplementary Table S1).

The interaction analysis results of capillary zone electrophoresis (CZE) showed that due to the different binding ratios, nCoV-S1-Apts formed stable polymorphic complexes with S1 (Fig. 1a), and had nanomolar affinity (*K*_D_ = 0.118 ± 0.033–85.610 ± 14.219 nM) (Fig. 1b). The interfering substances existing in vivo environments including human serum albumin (HSA), IgG Fc, normal human serum (NHS), and IgG Anti-S1 were introduced to evaluate the specificity of nCoV-S1-Apts. The results showed that the peak area of nCoV-S1-Apt1 did not change significantly during the interaction with the above interfering components (Fig. 1c and Supplementary Fig. S2). The results indicated that nCoV-S1-Apts exhibited stronger specificity and better anti-plasma protein interference properties, which is beneficial to reduce potential off-target effects and cytotoxicity (Supplementary Fig. S3). The affinity and specificity of nCoV-S1-Apt1 were verified by surface plasmon resonance (SPR) (Fig. 1d and Supplementary Fig. S4).

**Fig. 1:**
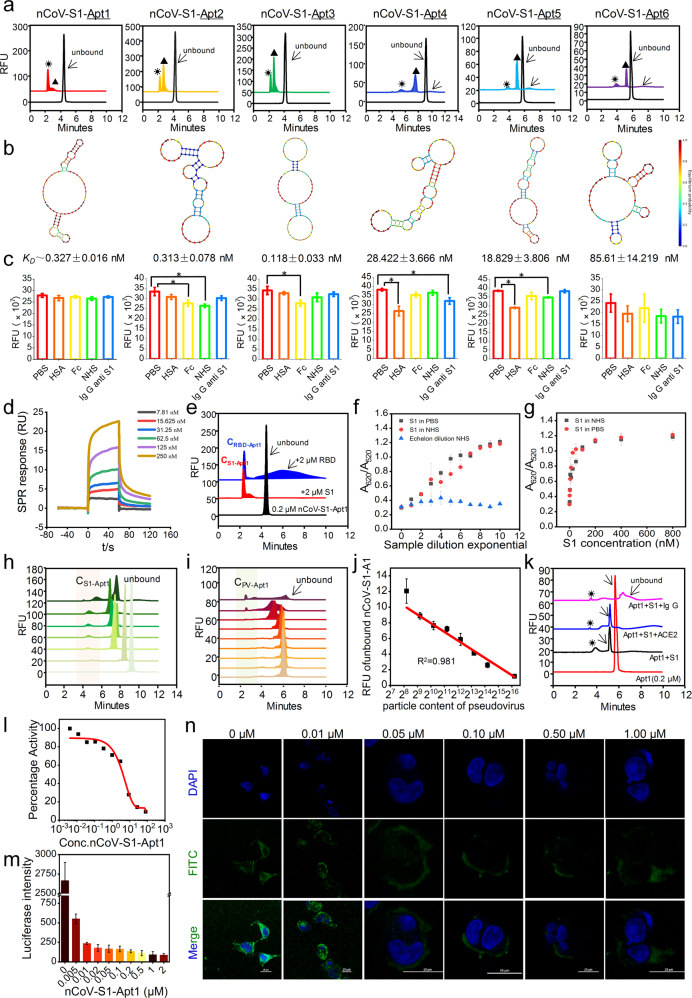
Identification of SARS-CoV-2-against aptamers with high neutralization activity. **a** The binding between aptamers of nCoV-S1-Apt1 ~6 (0.2 μM) and S1 protein (2 μM) was characterized by CZE. “☀” and “▲” indicate the two forms complex peaks formed by aptamer and S1 protein. The black arrows indicate the unbound aptamer peak. **b** The secondary structures and *K*_D_s of nCoV-S1-Apt1 ~6. **c** Specificity verification of aptamers of nCoV-S1-Apt1 ~6. The relative fluorescence unit (RFU) of 0.2 μM nCoV-S1-Apt1 ~6 peak areas in PBS (pH 7.2), HSA (2 μM), Fc (2 μM), NHS (20× diluted), and IgG anti-S1 (2 μM) were shown. *n* = 3; *p* < 0.05 (*). **d** Affinity characterization via surface plasmon resonance (SPR). **e** CE electrophoretogram of the binding of nCoV-S1-A1 to S1 (blue line) and RDB (red line), and the black line is the free nCoV-S1-Apt1 without adding protein. **f**, **g** Detection of S1 protein by AuNPs colorimetric assay using nCoV-S1-Apt1. R1, NHS was added for specific comparison by two-fold stepwise gradient dilution. R2 and R3, S1 at gradient concentrations (1.5625–800 nM) in PBS and 20× diluted NHS, respectively. **h** Detection of S1 protein with FAM-labeled nCoV-S1-A1 in NHS. Data from the bottom to the top are CE-analysis lines of 0.2 μM nCoV-S1-Apt1 incubated with S1 protein (0, 5, 10, 20, 50, 100, 200 nM) in 20× diluted NHS. C_S1-Apt1_ indicates the complex peaks formed by nCoV-S1-Apt1 and S1. **i** Detection of pseudovirus with FAM-labeled nCoV-S1-Apt1 in NHS. Data from the bottom to the top are the CE-analysis lines of 0.2 μM nCoV-S1-Apt1 incubated with various amounts of pseudovirus (0, 293, 586, 1172, 2344, 4688, 9375, 18750, 75,000 TU/ml) in 20× diluted NHS. C_PV-Apt1_ indicates the complex peaks formed by nCoV-S1-Apt1 and pseudovirus. **j** Standard curve of pseudovirus detection with nCoV-S1-Apt1 (*R*^2^ = 0.981). **k** Competition tests via CE. ACE2 protein or anti-S1 IgG compete with the nCoV-S1-Apt1 to bind S1 protein. 0.2 μM nCoV-S1-A1 (red line); 0.2 μM nCoV-S1-Apt1 + 0.2 μM S1 (black line); 0.2 μM nCoV-S1-Apt1 + 0.2 μM S1 + 0.4 ACE2 (blue line); 0.2 μM nCoV-S1-Apt1 + 0.2 μM S1 + anti-S1 IgG (pink line). **l** IC_50_ of nCoV-S1-Apt1 against RBD binding to ACE2 determined via competitive ELISA. **m** nCoV-S1-Apt1 inhibits the expression of luciferase by blocking pseudovirus infection. **n** CLSM images of GFP expression. Repression of GFP expression represents the pseudovirus infection is inhibited by nCoV-S1-Apt1. Cell nuclei were stained with DAPI (blue). Scale bar, 25 μm

In order to determine the possible binding site(s) of nCoV-S1-Apt1, we docked nCoV-S1-Apt1 to S1. Visualization of docking possess was performed using PyMOL. The results showed that nCoV-S1-Apt1 residues from 45 to 71 bind to the two main active sites designated for ACE2 binding (Supplementary Fig. S5). Different types of interactions were observed in the S1-aptamer complex, e.g., 32 polar contacts, 20 VdW, 15 H-bonds, 26 hydrophobic, 4 ionic, and 18 aromatic, yielding −12.17 kcal/mol binding free energy with *K*_D_ value of 0.1 nM. This data suggested that nCoV-S1-Apt1 may be used as a potent inhibitor of the RBD domain of S1.

In addition, the binding affinities between nCoV-S1-Apt1 and RBD were determined via CE (Fig. 1e). Data showed that nCoV-S1-Apt1 combined with RBD to form stable complexes with a *K*_D_ of 1.56 ± 0.22 μM. Therefore, we sought to determine the binding kinetics of nCoV-S1-Apts and S1. The *K*_D_ of nCoV-S1-Apts to S1 is 1.5 × 10^4^ times lower than that of RBD (Supplementary Fig. S6), which indicates that the binding time of nCoV-S1-Apts to S1 is significantly longer than that of RBD. S1 provides a more stable binding site for the aptamer than RBD, thereby greatly extending the binding time and exhibiting the kinetic advantage of drug-receptor interaction, and further helping to significantly improve the interaction potency.

To evaluate the recognition and detection potential of nCoV-S1-Apt1, we developed an AuNPs colorimetric assay, which could quickly and specifically detect S1 protein in human serum with a detection limit of ~3.125 nM (Fig. 1f, g and Supplementary Fig. S7). Meanwhile, FAM-labeled nCoV-S1-Apt1 was employed as a fluorescent probe to capture S1 protein in human serum (Fig. 1h). The CE laser-induced fluorescence (LIF) analysis results showed that nCoV-S1-Apt1 formed a significant complex with 5 nM of S1 protein in human serum. In addition, we introduced the SARS-CoV-2 spike-pseudotyped virus into the human serum, and tested it on CE-LIF. The results demonstrated that FAM-nCoV-S1-Apt1 exhibited satisfactory recognition (Fig. 1i) and detection linearity (Fig. 1j) in the range of 10^2^–10^5^ virus particles.

To confirm the multi-scale inhibitory effect of nCoV-S1-Apt1 on SARS-CoV-2, inhibition tests were carried out from 3 levels of RBD, S1, and pseudovirus. We established a ternary competitive binding assay, in which ACE2 protein or anti-S1 IgG was introduced to compete with the nCoV-S1-Apt1 (Fig. 1k). The results showed that the addition of ACE2 protein or anti-S1 IgG reduced the nCoV-S1-Apt1/S1 complex, suggesting that nCoV-S1-Apt1 competed with ACE2 protein or anti-S1 IgG to bind to S1 at a specific site. Furthermore, we evaluated the IC_50_ of nCoV-S1-Apt1 on the binding of RBD to ACE2 by competitive ELISA (Fig. 1l). nCoV-S1-Apt1 showed potent inhibition activity with IC_50_ of 80.12 nM.

To identify and verify the inhibitory effect and neutralization performance of nCoV-S1-Apt1 on S1/ACE2 binding and SARS-CoV-2 infection, we next employed a SARS-CoV-2 spike-pseudovirus infection model, which stably expresses both green fluorescent protein (GFP) and firefly luciferase. The data manifested that the introduction of nCoV-S1-Apt1 significantly reduced the expression of GFP and luciferase (Fig. 1m, n and Supplementary Fig. S8), and nCoV-S1-Apt1 displayed a dose-dependent inhibitory profile on pseudovirus infection. Therefore, nCoV-S1-Apt1 can prevent SARS-CoV-2 infection by binding to the RBD of S1 and hindering the recognition and interaction of S1 to ACE2, which implies its great potential as a new neutralizing antiviral agent against SARS-CoV-2 infection.

In summary, six DNA aptamers with high affinity to S1 protein were screened by CE-based SELEX in this study, and nCoV-S1-Apt1 showed excellent neutralization activity. Hence, this study proposes the potential use of aptamer in COVID-19 therapy or prevention, and provides a basis for the design of fusion inhibitor, neutralizing oligonucleotide, or targeted delivery system for SARS-CoV-2. It also presents an universal and feasible identification strategy for virus-suppressing aptamer selection based on multi-scale target selection. In addition, an authentic SARS-CoV-2 virus challenge and in vivo verification test should be performed in the next stage. We are working on it and digging in establishing an aptamer-based diagnosis and treatment platform for control of COVID-19.

## Supplementary information

Supplementary Materials
