# Supplementary material for: Identification of SARS-CoV-2-against aptamer with high neutralization activity by blocking the RBD domain of spike protein 1
Source: Signal Transduct Target Ther. 2021 Jun 10;6:227. doi: 10.1038/s41392-021-00649-6 (PMC8190169; doi:10.1038/s41392-021-00649-6)
Supplement: Supplementary file 1 — Supplementary Materials [file 41392_2021_649_MOESM1_ESM.docx]

**Supplemental Materials**

**Identification of SARS-CoV-2-against aptamer with high neutralization activity by blocking the RBD domain of spike protein 1**

Ge Yang ^1^, Ziyue Li ^1^, Irfan Mohammed ^1^, Liping Zhao ^1^, Wei Wei ^2^, Haihua Xiao ^3^, Weisheng Guo ^4^, Yongxiang Zhao ^5^, Feng Qu ^1^, and Yuanyu Huang ^1^

^1^ School of Life Science; Advanced Research Institute of Multidisciplinary Science; Institute of Engineering Medicine; Key Laboratory of Molecular Medicine and Biotherapy; Beijing Institute of Technology, Beijing 100081, China.

^2^ State Key Laboratory of Biochemical Engineering, Institute of Process Engineering, Chinese Academy of Sciences, Beijing 100190, China.

^3^ Beijing National Laboratory for Molecular Sciences, State Key Laboratory of Polymer Physics and Chemistry, Institute of Chemistry, Chinese Academy of Sciences, Beijing 100190, China.

^4^ Translational Medicine Center, Key Laboratory of Molecular Target & Clinical Pharmacology and the State Key Laboratory of Respiratory Disease, School of Pharmaceutical Sciences & the Second Affiliated Hospital, Guangzhou Medical University, Guangzhou 510260, China.

^5^ National Center for International Research of Biological Targeting Diagnosis and Therapy, Guangxi Key Laboratory of Biological Targeting Diagnosis and Therapy Research, Guangxi Medical University, Nanning 530021, Guangxi, China.

***** Correspondence: Yuanyu Huang, Email: [yyhuang@bit.edu.cn](mailto:yyhuang@bit.edu.cn); Tel: +86-10-68911089.

**Content**

[I. Method and materials 3](#_Toc65310721)

[1 Materials 3](#_Toc65310722)

[2 CE-SELEX 3](#_Toc65310723)

[2.1 Capillary electrophoresis conditions 3](#_Toc65310724)

[2.2 Co-incubation of target protein and ssDNA library 4](#_Toc65310725)

[2.3 Separation and collection of complexes 4](#_Toc65310726)

[2.4 Sub-library amplification and purification 4](#_Toc65310727)

[3 Aptamer performance evaluation 5](#_Toc65310728)

[3.1 Affinity evaluation and dissociation constant measurement 5](#_Toc65310729)

[3.2 Specificity evaluation 5](#_Toc65310730)

[4 Cell viability 6](#_Toc65310731)

[5 Affinity determination with surface plasmon resonance (SPR) 6](#_Toc65310732)

[6 Molecular docking 7](#_Toc65310733)

[7 Comparison of affinities of aptamer to S1 and RBD 7](#_Toc65310734)

[8 Aptamer-facilitated detection of S1 and pseudovirus in normal human serum 7](#_Toc65310735)

[8.1 AuNPs colorimetric assay based on aptamer 7](#_Toc65310736)

[8.2 nCoV-S1-A1-facilitated pseudovirus detection via CE-LIF 8](#_Toc65310737)

[8.3 Competitive ELISA 8](#_Toc65310738)

[9 Neutralization activity of nCoV-S1-A1 evaluated with ACE2-decorated cell line 9](#_Toc65310739)

[9.1 Confocal imaging 9](#_Toc65310740)

[9.2 Luciferase activity determination 9](#_Toc65310741)

[10 Statistical analysis 10](#_Toc65310742)

[II. Extended figures and table 11](#_Toc65310743)

[1 Supporting Figs. S1, S2, S3, S4, S5, S6, S7 and S8 11](#_Toc65310744)

[2 Supporting Table S1 14](#_Toc65310745)

[III Reference 15](#_Toc65310746)

# I. Method and materials

# 1 Materials

H_3_BO_3_, Na_2_B_4_O_7_ and NaOH were purchased from [Beijing Beihua Fine Chemicals Co., Ltd.](https://dict.cn/BEIJING%20BEIHUA%20FINE%20CHEMICALS%20CO_2E%2C%20LTD_2E%20%28BEIJING%20CHEMICAL%20WORKS%29) Taq PCR Master Mix, dd H_2_O, Nuclelc Acid Dye Gene Green, 6 × DNA Loading Buffer, 50 bp DNA Ladder and Realtime PCR Master Mix were purchased from Tiangen Biotech (Beijing) Co., Ltd.

The single-stranded DNA (ssDNA) library contains a 40-base random region flanked by two 20-base primer regions (5'-FAM-AGC AGC ACA GAG GTC AGA TG-(N40)-CCT ATG CGT GCT ACC GTG AA-3'). The 5’ terminal was labeled with 6-carboxyfluorescein (FAM). The forward and reverse primer sequences are as follow: P: 5’-AGC AGC ACA GAG GTC AGA TG-3’, P':5’-CCT ATG CGT GCT ACC GTG AA-3’, respectively. The fluorescently labeled ssDNA was purified by reversed-phase HPLC. All DNA libraries, PCR primers, and aptamers were obtained from Biological Engineering Technology and Services, Shanghai, China.

S1, RBD, Fc and IgG anti S1, was purchased from Sino Biological Inc. Normal human serum was obtained from Beijing BioDee Biotechnology Co., Ltd. Human albumin was obtained from Sigma-Aldrich (St. Louis, MO).

Luciferase Assay System and Passive Lysis Buffer (PLB) were bought from Promega Co. Ltd. (Madison, USA). Dimethyl sulfoxide (DMSO) and 3-[4,5-dimethylthiazol-2-yl]-2,5-diphenyl tetrazolium bromide (MTT) were bought from Sigma-Aldrich (St. Louis, MO). The cell culture reagents including trypsin, EDTA, penicillin–streptomycin, Opti-MEM, Dulbecco’s modified Eagle’s medium (DMEM), fetal bovine serum (FBS) were purchased from Thermo Fisher.

Unless otherwise noted, all samples and buffers were prepared in ultrapure water.

# 2 CE-SELEX

## 2.1 Capillary electrophoresis conditions

Beckman P/ACE MDQ (Beckman-coulter, Fullerton, CA, USA) equipped with a LIF detector was used for protein and ssDNA library mixture separation and protein–ssDNA complex collection, by which FAM-labeled ssDNA was detectable. All capillary electrophoresis (CE) data were analyzed using 32 Karat software. The separation was conducted by an uncoated fused silica capillary (75 μm i.d. × 50.2 cm (40.0 cm effective), Sino Sumtech, Handan, Hebei, China) at room temperature. Samples were injected into the capillary under the pressure of 0.5 psi for 5 s, and a running voltage of 15 kV (375 V/cm) or 20 kV (498 V/cm) was employed to drive separation. Excitation was generated using the 488 nm line of an Ar^+^ laser (Beckman Coulter) and emission was collected at 520 nm. Running buffer was composed of 50 mM H_3_BO_3_ / Na_2_B_4_O_7_（pH 7.8）unless otherwise stated. To remove any possible adsorption of proteins on the inner surface of the capillary and to maintain reproducible CE separation, the capillaries were treated with 0.1 M NaOH for 3 min, followed by water for 3 min and running buffer for 3 min after every consecutive sample injections.

## 2.2 Co-incubation of target protein and ssDNA library

To place ssDNA in their desired conformation, 100 μM of ssDNA library was treated at 94 °C for 5 min, followed by cooling slowly to 4 °C before use. To form complexes, the appropriate volumes of ssDNA and protein stock solutions were mixed and diluted with incubation buffer to obtain the desired concentrations of ssDNA and proteins. The final sample solution was 10 μL. Before injection into CE, the sample solution was incubated at 37 °C for 15 min.

## 2.3 Separation and collection of complexes

The first round of CE-SELEX is positive screening, 0.2 μM of ssDNA library was incubated with 0.1 μM S1 protein at 37 °C for 15 min on heating block. The incubation mixture was analyzed by nonequilibrium capillary electrophoresis of equilibrium mixtures (NECEEM) method, and the collection period of complex components was 2.8 to 3.6 min (Fig. S1a). The second round was negative screening. The recombinant S1 protein contained Fc fragment, thus Fc fragment was introduced as the negative target protein to eliminate the Fc-bound ssDNA from the sub-library in the previous round. The Sub-ssDNA library was incubated with 2μM of Fc fragment, which was a high target input concentration that can eliminate ssDNA to the greatest extent. The collection period of unbound ssDNA was 5.9 to 6.8 min (Fig. S1b). The third round of CE-SELEX was carried out in 20 × diluted human serum, 0.04 μM of S1 protein was incubated with the sub-library obtained from round 2, and the collection period was 3.1 to 4.0 min (Fig. S1c).

## 2.4 Sub-library amplification and purification

The target-bound ssDNAs were amplified by routine PCR. The reverse primer was labeled by FAM for sub-library purification. The PCRs were set up in a final volume of 50 μL with 2 μL of template described as above, 275 μL of 2 × Taq PCR MasterMix, 0.4 μM of P1 and 0.4 μM of P2. The reaction was performed at 94 °C for 3 min; then 25 cycles of 94 °C for 30 s, 60 °C for 30 s and 72 °C for 30 s; and at 72 °C for 3 min for extension finally. Asymmetric PCR was used to prepare secondary library. Herein, using the above conventional PCR products as templates, perform asymmetric PCR amplification to prepare sub-ssDNA library. Optimized asymmetric PCR conditions were as follow: total volume was 100 μL, of which 2 × Taq Plus PCR Master Mix was 50 μL, primer P1 was 1.8 μmol/L, and primer P2 was 60 nmol/L. PCR conditions were as follow: pre-denaturation at 94 °C for 1 min, denaturation at 94 °C for 30 s, annealing at 60 °C for 30 s, extension at 72 °C for 30 s, extension at 72 °C for 5 min after 30 cycles. After PCR or asymmetric PCR, the product needs to be concentrated and purified to obtain a template for asymmetric PCR or a secondary library for the next round of screening. Concentration and purification methods were as follow: the PCR product was electrophoresed on a 2% (w/v) agarose gel, 60 V, 40 min; the desired gel was transferred to a 1.5 mL centrifuge tube and centrifuged at 12000 r/min for 1 min. Freezed at -20 °C for 20 min. The frozen gel was immediately mashed, then 500 μL of water was added and shaken 200 times; 500 μL of Tris saturated alcohol phenol was added to the tube, shaken 200 times, and centrifuged at 4 °C 12000 r/min for 15 min. The supernatant was added to a 1.5 mL centrifuge tube containing 500 μL of chloroform:isoamyl alcohol (v/v, 24:1), shaken 200 times, and centrifuged at 4 °C, 12000 r/min for 15 min. After discarding the supernatant, the precipitate was placed on a clean absorbent paper and dried to obtain ssDNA.

# 3 Aptamer performance evaluation

## 3.1 Affinity evaluation and dissociation constant measurement

High-throughput sequencing of aptamer candidates was performed by sangon biotechnology (Table S1), and the secondary structures was analyzed by NUPACK (<http://www.nupack.org/>). 0.2 μM of aptamer candidates (nCoV-S1-Apt1 ~ Apt6) were incubated with S1 protein. According to the non-equilibrium capillary electrophoresis (NECEEM) of the equilibrium mixture, the dissociation constant (*K_D_*) was calculated according to the following formula:

$$\text{K}_{\text{D}}\text{=}\frac{\left[ \text{P} \right]_{\text{0}}\left( \text{1+}\frac{\text{A}_{\text{1}}}{\text{A}_{\text{2}}\text{+}\text{A}_{\text{3}}} \right)\text{-}\left[ \text{DNA} \right]_{\text{0}}}{\text{1+}\frac{\left( \text{A}_{\text{2}}\text{+}\text{A}_{\text{3}} \right)}{\text{A}_{\text{1}}}}$$

*[P]_0_*: protein concentration; *[DNA]_0_*: aptamer concentration; *A1*: peak area of free aptamer; *A2*: peak area of protein-aptamer complex; *A3*: peak area of complex distribution region.

## 3.2 Specificity evaluation

0.2 μM of aptamer candidates (nCoV-S1-Apt1 ~ Apt6) were incubated with PBS (pH 7.2), HSA(2 μM), Fc(2 μM), normal human serum (20 times dilution) and Ig G anti S1 (2 μM), respectively, at 37 °C for 15 min. The mixture was analyzed by capillary zone electrophoresis (CZE) to calculate the peak area of the aptamer candidates.

The cross reactivity of the aptamer (nCoV-S1-Apt1) to interfering substances (human serum albumin (HSA), IgG Fc, IgG Anti-S1 and normal human serum (NHS)) were further tested at different concentrations. 0, 0.5, 1, 2, 5, 10 μM of HSA, IgG Fc, IgG Anti-S1 and NHS with dilution ratios of 10, 20, 30, 40, 50 were used to interact with the nCoV-S1-Apt1 (FAM labeled), and the results in Fig. S2.

# 4 Cell viability

Cells were seeded into 96-well plate (1×10^4^ cells per well) and cultured at 37 °C in a humidified atmosphere containing 5% CO_2_ for 12 h. Then, cells were incubated with multiple concentrations of nCoV-S1-Apt1 (62.5, 125, 250, 500, 1000 nM, respectively), remdesivir (62.5, 125, 250, 500, 1000, 2000 nM, respectively), chloroquine (62.5, 125, 250, 500, 1000, 2000 nM, respectively)^1^ for 24 h. No aptamer or drugs were used in mock group. Then, each well was added with MTT solution (10 μL, 5 mg/mL) and incubated at 37 °C for 4 h. After incubation, DMSO was used to dissolve formazan crystals in each well. The multimode microplate reader was used to measure formazan absorbance at 540 nm, and the absorbance of 650 nm was also measured as a reference wavelength. Finally, cell viability of each group was calculated as follows:

$$\text{C}\text{ell viability}\left( \text{\%} \right)\text{=}\frac{\text{OD540}\left( \text{sample} \right)\text{-OD650(sample)}}{\text{OD540}\left( \text{mock} \right)\text{-OD650(mock)}}\text{×100}$$

The results were shown in Fig. S3.

# 5 Affinity determination via surface plasmon resonance (SPR)

The affinity between nCoV-S1-Apt1 and S1 was also determined with surface plasmon resonance (SPR), and small molecule of chloroquine was introduced as a control. S1 protein in acetate buffer (pH 5.5) was injected into the sensor chip to reach ∼1000 RU immobilization level. The deactivation was done by ethanolamine-HCl to block unreacted carboxyl groups. The binding analysis was carried out with the aptamer nCoV-S1-Apt1 (7.81, 15.625, 31.25, 62.5, 125, 250 nM) and chloroquine (0.78125, 1.5625, 6.25, 12.5, 25, 50 µM) at different concentrations using a Biacore T200 instrument (GE Healthcare). PBS and 0.005% tween-20 solution mixture was used as the running buffer, and 50 mM NaOH as the regeneration buffer. Upon injection of nCoV-S1-Apt1 or chloroquine, sensorgrams recording the association/dissociation behavior of the S1-aptamer or S1-chloroquine complex were collected. By varying the nCoV-S1-Apt1 or chloroquine concentration, a series of sensorgrams were obtained (Fig. S4) and subsequently analyzed using the 1:1 Langmuir model provided in the BIAevaluation software (version 4.1) to calculate the equilibrium dissociation constant *K_D_*.

# 6 Molecular docking

The 3D structure of S1 protein was retrieved from RCSB PDB under ID of 6VSB, visualized and prepared by UCSF Chimera. Then, all the water molecules were removed from the complex structure. The 3D structure of ssDNA aptamer was initially prepared by using RNA composer, converted into DNA 3D coordinates by Mode RNA webserver and MDWeb webserver. Binding residues of S1 protein were predicted by literature and from Uniprot knowledge base database (Fig. S5). The complex structure between the S1 protein and the S1-aptamer molecule was predicted using hybrid protein-DNA docking algorithm, HDOCK. Specifically, given the individual structure of the protein and the S1-aptamer molecule, HDOCK used a fast Fourier transform (FFT)-based search strategy to globally sample all possible binding modes between the two proteins. Then, all the sampled binding modes were evaluated by iterative knowledge-based scoring function ITScorePP. Last, the binding modes were ranked according to their binding energy scores, and the top ten binding modes were provided. During the docking calculation, all the default parameters were used. Namely, the grid spacing was set to 1.2 Å for 3D translational search, the angle interval was set to 15 for rotational sampling in 3D Euler space, and the binding interface information in the PDB was automatically applied during the template-based modeling of individual structures. The binding residues analysis were performed by OrppegioWeb webserver. In addition, binding free energy of protein-aptamer complex was calculated by MD simulations.

# 7 Comparison of affinities of aptamer to S1 and RBD

CE analysis was performed after co-incubation of 0.2 μM of nCoV-S1-A1 with 2 μM of S1 and 2 μM of RBD, respectively. The evaluation method of affinity was described in section of 3.1. The data were shown in Fig. S6.

# 8 Aptamer-facilitated detection of S1 and pseudovirus in normal human serum

## 8.1 AuNPs colorimetric assay based on aptamer

**Principle:** Gold nanoparticles (AuNPs) have characteristics of high extinction coefficient and strong inter-particle effect. 10 nm AuNPs showed a wine-red color in the dispersion state and a characteristic light absorption at 520 nm. After the addition of NaCl solution, the electrostatic repulsion between exposed AuNPs was destroyed and coagulated, gradually turning to purple and blue. The light absorption decreased at 520 nm and increased at 600-700 nm. When AuNPs were coated with aptamers, the AuNPs were protected by aptamer and would not coagulate due to the introduction of NaCl. However, the introduction of the target protein competed the aptamers from the AuNPs, so that the AuNPs lose the protection of the aptamers and become exposed. Thus, the introduction of NaCl causes the coagulation. The degree of color change of AuNPs is proportional to the concentration of the introduced target protein. Therefore, the concentration of the target protein can be qualitatively or quantitatively detected by macroscopic observation or determination of the absorption value (A620/520) of the solution.

**Preparation of AuNPs:** AuNPs were synthesized through the following classical citrate reduction method. Briefly, 100 mL 1 mmol/L HAuCl_4_ solution was heated to boiling. Then, 2 mL of 194 mmol/L sodium citrate solution was rapidly injected under synchronous stirring. After boiling for 15 min, the reaction flask was taken out and cooled slowly to room temperature.

**Colorimetric analysis:** In 96-well plate, 25 μL of AuNPs solution was incubated with an equal volume of aptamer solution (4 μmol/L) for 10 min. Then, 25 μL of S1 with gradient concentrations (1.5625 nM ~ 800 nM) in PBS or 20 × diluted normal human serum were added and incubated at room temperature for 15 min. Normal human serum was also added for specific comparison by two-fold stepwise gradient dilution. Then, the color change of AuNPs solution was observed after 10 μL of NaCl solution (900 mmol/L) was added. Finally, the absorbance at 620 nm and 520 nm wavelength was recorded with a microplate reader. The optical observation was shown in Fig. S7.

## 8.2 nCoV-S1-A1-facilitated pseudovirus detection via CE-LIF

SARS-CoV-2 pseudovirus were obtained from Fubao Biotechnology Co., Ltd., which were constructed by using a retrovirus preparation system. The membrane surface of the pseudovirus displays the spike protein of SARS-CoV-2. Pseudovirus at the concentrations of 75000 TU/ml, 18750 TU/ml, 9375 TU/ml, 4688 TU/ml, 2344 TU/ml, 1172 TU/ml, 586 TU/ml, and 293 TU/ml were respectively incubated with 0.2 μM of nCoV-S1-A1 in 20 × diluted normal human serum, and then analyzed by CE.

## 8.3 Competitive ELISA

SARS-CoV-2 inhibitor screening ELISA Kit (KIT001) was purchased from Sino Biological Inc. The SARS-CoV-2 inhibitor in the samples competes with ACE2-His (Cat: 10108-H08B) to bind with immobilized S Protein RBD of SARS-CoV-2. The signal color becomes lighter as the concentration of SARS-CoV-2 inhibitor increases. The aptamer inhibitory activity was measured in strict accordance with the experimental instructions of Kit.

# 9 Neutralization activity of nCoV-S1-A1 evaluated with ACE2-decorated cell line

The neutralization activity of nCoV-S1-Apt1 was evaluated by using ACE2-stably expressing cell line and SARS-CoV-2 pseudovirus.

## 9.1 Confocal imaging

Hek293T/ACE2 cell stably expressing ACE2 was prepared by pAA V-IRES-SARS-CoV-2 S transfection via PolyJet (SignaGen, USA), which was used in the pseudovirus infection/neutralization assays. RNA sequences of green fluorescent protein (GFP) and firefly luciferase were incorporated in SARS-CoV-2 pseudovirus. The infection efficiency was determined by observing the expression of GFP and detecting the activity of luciferase. The Hek293T/ACE2 cells were inoculated into the 96-well plate (1 × 10^4^ cells per well), and the pseudovirus infection was carried out 24 h later. The frozen pseudovirus were taken out and melted on the ice. In aptamer inhibits pseudovirus infection assays, 8 μL of nCoV-S1-Apt1 (final concentration: 0, 0.1, 0.2, 0.5, 1, 2 μM) were added to the Hek293T/ACE2 cells together with 2 μL of pseudovirus. Fresh culture medium was replaced to continue the culture after 6 h. After 60 h, the expression of GFP was examined by Confocal laser scanning microscope (CLSM) (Fig. S8), and luciferase activity was analyzed to determine the pseudovirus infection efficiency.

## 9.2 Luciferase activity determination

The Hek293T/ACE2 cell was subjected the same treatment with Confocal imaging assay. In addition to the concentrations used in Confocal observation (0, 0.1, 0.2, 0.5, 1, 2 μM), lower dose of aptamer was used in this assay (0.005, 0.01, 0.02, 0.05 μM). The pseudovirus-infected cells were washed lightly with pre-cooled 1 × PBS. Then 50 μL of 1 × Passive Lysis Buffer (PLB) was added into each well, and then the cells were shaken for 20 min at 25 °C to enable cells to decompose adequately. The cell lysates were centrifuged at 12, 000 rpm for 30 s at 25 °C. Finally, 5 μL of supernatant was transferred into the 96-well detection plate, 50 μL of substrates were added to each well, and the luciferase activity was measured by multimode microplate reader.

# 10 Statistical analysis

Significance analysis was performed by Origin 8.0 software. All data are shown as the mean ± SD, and differences with p < 0.05 were considered significant (defined as *p < 0.05). IC50 values were calculated using the competitive ELISA standard curve fitting (Graphpad Software, La Jolla, CA, USA).

# II. Extended figures and table

# 1 Supporting Figs. S1, S2, S3, S4, S5, S6, S7 and S8

Fig. S1. CE-based SELEX screening of S1-against aptamers. a. Round 1. b. Round 2. c. Round 3.

Figure S2. Specificity verification of nCoV-S1-Apt1 aptamer. The relative fluorescence unit (RFU) of nCoV-S1-Apt1 (0.2 μM) peak area was shown in the figure. Human serum albumin (HSA), IgG Fc and IgG Anti-S1 were used at the concentrations of 0, 0.5, 1, 2, 5, and 10 μM. Normal human serum (NHS) was diluted at various times (10 ×, 20 ×, 30 ×, 40 ×, 50 ×). n = 3.

Fig. S3. Cytotoxicity of nCoV-S1-A1, remdesivir and chloroquine evaluated by MTT. a. nCoV-S1-A1; b. Radecivir; c. Chloroquine.

Fig. S4. Characterization of the affinity between nCoV-S1-Apt1 and S1 protein with surface plasmon resonance (SPR). Chloroquine was introduced as control. a. nCoV-S1-Apt1; b. chloroquine. The nCoV-S1-Apt1 showed a high affinity for S1 protein (*K_D_* ~ 49.71 nM), while chloroquine had a very weak binding to S1 protein (*K_D_* ~ 17.04 μM).


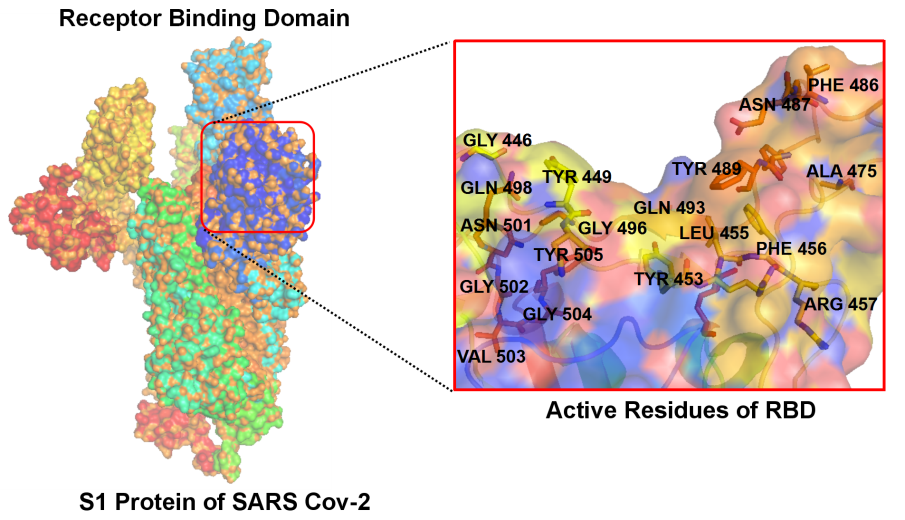


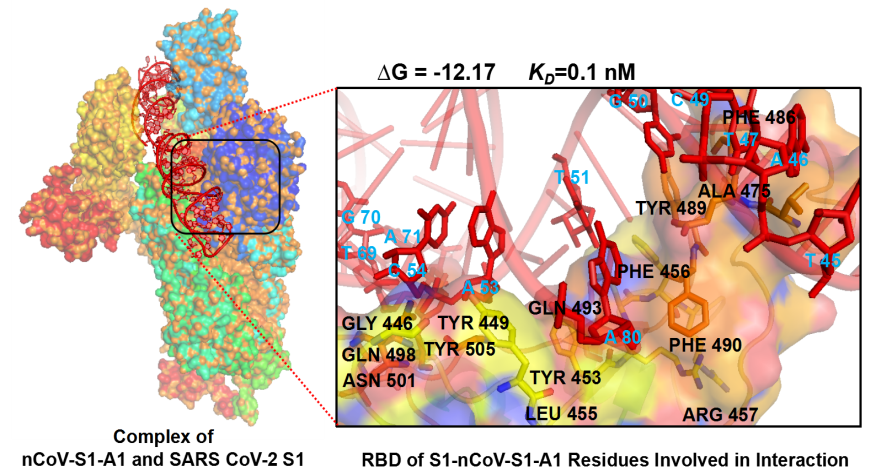


Fig. S5. Binding sites predicted by molecular docking. The active site of S1 protein is shown with residue names and identifier.

Fig. S6. *K_D_s* of nCoV-S1-A1 to S1 and RBD.


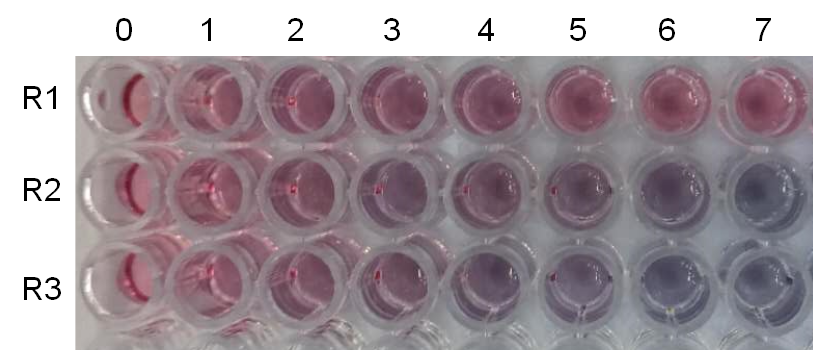


Fig. S7. Detection of S1 protein in human serum with AuNPs colorimetric assay.

Fig. S8. CLSM images of nCoV-S1-A1 inhibits pseudovirus infection. Expression of green fluorescent protein (green). Scale bar, 100 μm.

# 2 Supporting Table S1

Table S1. Aptamer candidates with the highest enrichment abundance.

| Nam. | Aptamers sequences(5'-3') | Freq. | Tm(℃) | ΔG  (kcal/mol at 37 °C) |
| --- | --- | --- | --- | --- |
| nCoV-S1-A1 | P1-CCGCAGGCAGCTGCCATTAGTCTCTATCCGTGACGGTATG-P2 | 280 | 62.5 | -7.29 |
| nCoV-S1-A2 | P1-GCAGCTAAGCAGGCGGCTCACAAAACCATTCGCATGCGGC-P2 | 54 | 48.9 | -3.07 |
| nCoV-S1-A3 | P1-GGGAATGCTTGTGGAGATGAACACGCCATTACTGCCGTAC-P2 | 54 | 54.6 | -2.49 |
| nCoV-S1-A4 | P1-GCGAAGCGTACCGGCTACCCAGTGACAGTCGCCGTGGGTC-P2 | 50 | 50.3 | -2.45 |
| nCoV-S1-A5 | P1-GCCACATTAGTCTCACCACTACCTGCGTACCTACCGCCGC-P2 | 37 | 43.6 | -1.67 |
| nCoV-S1-A6 | P1-CGACTTGCCTATCGGCATGACACAATCTTTTGGAGCGTAA-P2 | 35 | 46.5 | -1.54 |

P1 and P2 were the fixed primer sequence at terminals of the ssDNA library sequences. P1: 5'- AGCAGCACAGAGGTCAGATG-3'; P2: CCTATGCGTGCTACCGTGAA-3'.

# III Reference

1 Xue, J. et al. Chloroquine is a zinc ionophore. *PLoS One* **9**, e109180, (2014).
